# Experiences of self-harm and suicidality in a community sample of Irish Travellers

**DOI:** 10.1192/j.eurpsy.2023.2372

**Published:** 2023-07-19

**Authors:** R. Mcmanus, M. McGovern, K. Tong, J. O’ Brien, A. Doherty

**Affiliations:** 1 Cheeverstown House, Dublin, Ireland; 2 Te Whatu Ora Health, Wellington, New Zealand; 3 Central Mental Hospital; 4 Exchange House Ireland; 5 University College Dublin, Dublin, Ireland

## Abstract

**Introduction:**

Irish Travellers are an indigenous minority in Ireland with distinct history and culture, of which nomadism was traditionally an important feature. Travellers experience disadvantages in education, employment, housing and health. Suicide is a big problem in the Traveller community, their suicide rate is 11%: 6 to 7 times higher than the general population. 59.4% of Traveller men and 62.7% of Traveller women reported that their mental health has not been good for one or more days in the last 30. Despite the high rates of suicide, there is a paucity of clinical research into mental health of Travellers.

**Objectives:**

Aim was to improve the scientific knowledge of the mental health of Irish Travellers by addressing the gap in the scientific literature.

The objective was to conduct a qualitative assessment of Travellers who have experienced self-harm and suicidal thoughts themselves, or who have a family member who has experienced same, by exploring their personal experience of distress, adversity and illness.

**Methods:**

We conducted semi-structured interviews exploring the following topics: self-harm, bereavement by suicide, experience of mental illness and of seeking treatment, stigma, discrimination and perceptions of research participation. Participants were recruited from community Traveller organisations in order to improve participation. Data were analysed using Nvivo software for thematic analysis.

**Results:**

Our participants aged from 22-62. 67% reported a personal history of self-harm, 83% had a psychiatric diagnosis. None were actively engaged with a Psychiatric team.

The main themes from the data were discrimination, identity issues, cultural understanding in healthcare settings, mental health and wider societal issues. Our findings showed that many Travellers who suffer from mental health problems and suicidal thoughts, find it hard to discuss problems openly within their families and communities due to stigma and shame, despite the high incidence of suicide. Many reported experiencing idenity crises, and a sense of not belonging in society, particularly since the introduction of legislation preventing them from aspects of their traditional lifestyles. Other common topics were literacy issues, womens and LGBTQplus rights.

**Conclusions:**

Travellers are a marginalised group in our society with high rates of socioeconomic deprivation, which we know is a factor in mental illness and thoughts of self-harm or suicide. There is a need for improved education for mental healthcare workers into the culture of Travellers and for increased sensitivity and awareness of how to engage with patients with literacy issues. Stigma remains an issue within the Travelling community and more work needs to be done to improve engagement between Travellers and mental health services in order to prevent acute mental health crises and/or suicidal behaviour. Travellers remain a difficult to reach and under-researched group in our society.

**Disclosure of Interest:**

None Declared

